# Entropy Measures Quantify Global Splicing Disorders in Cancer

**DOI:** 10.1371/journal.pcbi.1000011

**Published:** 2008-03-14

**Authors:** William Ritchie, Samuel Granjeaud, Denis Puthier, Daniel Gautheret

**Affiliations:** 1 Université de la Méditerranée, INSERM ERM 206, Technologies Avancées pour le Génome et la Clinique, Marseille, France; 2 Université Paris-Sud 11, CNRS UMR 8621, Institut de Génétique et Microbiologie, Orsay, France; University of California Santa Cruz, United States of America

## Abstract

Most mammalian genes are able to express several splice variants in a phenomenon known as alternative splicing. Serious alterations of alternative splicing occur in cancer tissues, leading to expression of multiple aberrant splice forms. Most studies of alternative splicing defects have focused on the identification of cancer-specific splice variants as potential therapeutic targets. Here, we examine instead the bulk of non-specific transcript isoforms and analyze their level of disorder using a measure of uncertainty called Shannon's entropy. We compare isoform expression entropy in normal and cancer tissues from the same anatomical site for different classes of transcript variations: alternative splicing, polyadenylation, and transcription initiation. Whereas alternative initiation and polyadenylation show no significant gain or loss of entropy between normal and cancer tissues, alternative splicing shows highly significant entropy gains for 13 of the 27 cancers studied. This entropy gain is characterized by a flattening in the expression profile of normal isoforms and is correlated to the level of estimated cellular proliferation in the cancer tissue. Interestingly, the genes that present the highest entropy gain are enriched in splicing factors. We provide here the first quantitative estimate of splicing disruption in cancer. The expression of normal splice variants is widely and significantly disrupted in at least half of the cancers studied. We postulate that such splicing disorders may develop in part from splicing alteration in key splice factors, which in turn significantly impact multiple target genes.

## Author Summary

RNA splicing is the process by which gene products are pieced together to form a mature messenger RNA (mRNA). In normal cells, RNA splicing is a tightly controlled process that leads to production of a well-defined set of mRNAs. Cancer cells, however, often produce aberrant, mis-spliced mRNAs. Such disorders have not been quantified to date. To this end, we use a well-known measure of disorder called Shannon's entropy. We show that overall splicing disorders are highly significant in many cancers, and that the extent of disorder may be correlated to the level of cell proliferation in each tumor. Surprisingly, genes that control the splicing mechanism are unusually frequent among genes affected by splicing disorders. This suggests that cancer cells may withstand harmful chain reactions in which splicing defects in key regulatory genes would in turn cause extensive splicing damage. As mis-spliced mRNAs are widely studied for cancer diagnosis, awareness of these global disorders is important to distinguish reliable cancer markers from background noise.

## Introduction

The majority of mammalian genes produce alternative transcripts as part of their normal expression program [1]–[4]. Alternative transcripts include splicing, polyadenylation and transcription initiation variants which can be expressed differentially in different tissues [4]–[7] providing the fine tuning of gene expression required for cell differentiation and tissue-specific functions. Disruptions in the balance of alternative transcripts, especially at the splicing level, are known to affect angiogenesis [8], cell differentiation [9] and invasion [10]. A large body of evidence has established connections between alternative splicing defects and cancer, so that the identification of transcript isoforms is now considered an important avenue in cancer diagnosis and therapy [11],[12].

The disruption of splicing isoform expression in cancer may result from very different underlying genetic events. On one hand, mutations in cis-regulatory sequences lead to the abnormal expression of specific isoforms, as observed for example in the BRCA1 gene in breast and ovarian cancer [13]. Another class of event includes alterations of the mRNA processing machinery or its signalling pathway. These may affect the splicing of specific genes such as CD44 [14]–[16], but may also cause wider perturbations of isoform expression as the processing of multiple genes can be simultaneously affected [17]–[20]. Evidence for wider changes in alternative transcription linked with cancer are present for instance in EST databases, where a large fraction of splice variant are actually tumor-specific [21]. However, while most studies of splicing and cancer attempt to isolate “signature” splice variants with significant over-expression in disease cells, no published work to date has focused on the bulk of splicing disruption that potentially arises when the splicing machinery is impaired.

The aim of the present study is to evaluate the extent and modalities of non-specific alternative transcript disruptions in cancer. Instead of seeking “interesting” signature isoforms, we analyzed the distribution of all isoforms from a single gene in a given tissue. We postulated that, in a tissue where the splicing machinery is impaired, the distribution of isoforms may be more disordered than in a control tissue. To measure the level of disorder in cDNA and cDNA tag libraries, we borrowed the notion of entropy from information theory. We applied this measure to all three types of alternative transcription, comparing isoform distributions in pairs of disease and normal tissues. Our results show that neither alternative polyadenylation nor alternative transcription initiation are associated with a disordered isoform expression. However, in half of the cancers studied, alternative splicing showed a highly significant entropy gain relative to the corresponding normal tissues. We analyze this entropy gain and discuss its possible causes.

## Results

### Isoform Entropy: Definition

**Figure 1:**
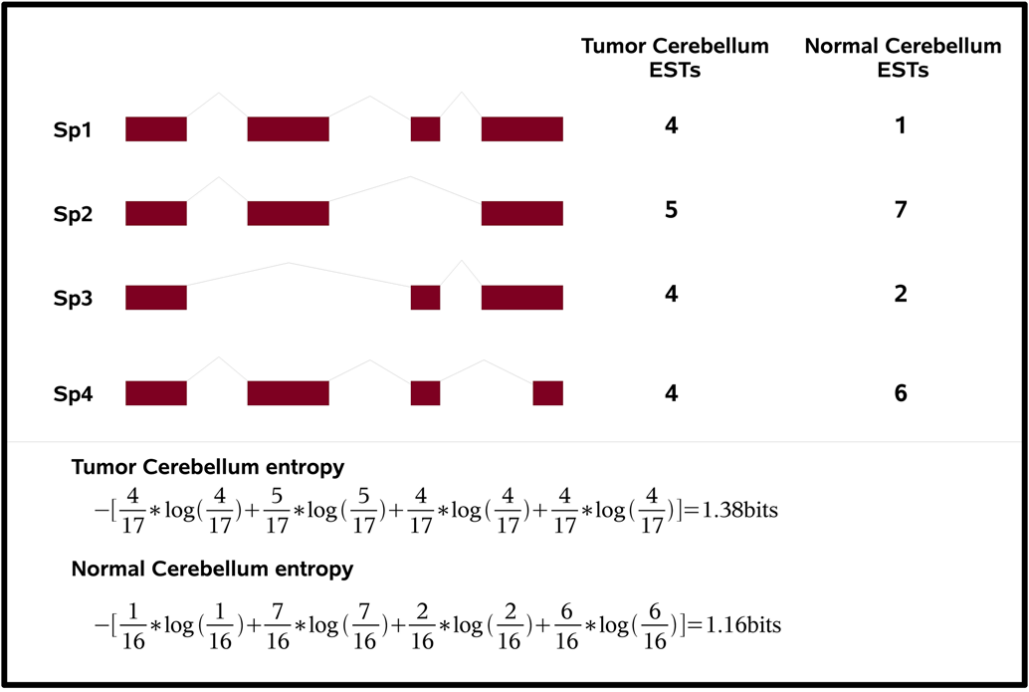
Example of Shannon's entropy calculation for a gene with four splicing isoforms SP1‥SP4. EST counts are provided for each isoform in a normal and cancer tissue. In this example, isoform entropy is higher in the cancer tissue (1.38 versus 1.16 bits).

Given a random variable *X* with probabilities *P*(*x_i_*) for discrete set of events *x1*,….,*k*, Shannon's entropy, also known as Information Entropy, is defined by:
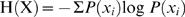
The entropy, and thus the disorder, is maximal when the probability of all the events *P*(*x_i_*) are equal and thus the outcome is most uncertain. Here, Shannon's entropy is applied to the expression profiles of different transcript isoforms for a given context. In the Figure 1 example, Gene1 has 4 alternative splice forms (*SP1*…*SP4*) and we are interested in their expression in normal cerebellum and cerebellum tumor tissues. For each splice form, we count the number of transcripts observed in different tissue types (for instance ESTs/cDNAs matching splice form *SP1* are observed 4 times in cerebellum tumor libraries and once in normal tissue libraries). For this gene, isoform entropy across the four splice forms is higher in tumor than in normal cerebellum tissues, reflecting a more uniform tissue distribution of isoforms in the tumor libraries.

### Cancer Tissues Have Higher Splicing Isoform Entropy

We hypothesised that impairment of the transcriptional or post-transcriptional control machinery in cancer or other diseases should result in the loss of a tissue-specific expression pattern of certain transcript isoforms. This loss can be measured by a gain of entropy in the expression pattern of isoforms of a given gene. By averaging entropy gains or losses on a sufficient number of genes expressed in a disease/normal tissue pair, we should observe a significant entropy bias if isoform expression is altered in this disease.

We obtained transcript isoform collections from the FANTOM3 database [1] for initiation variants and the ATD database [22] for polyadenylation and splicing variants. We then related isoforms to cDNA or cDNA tag counts and mapped each cDNA or tag to its tissue/disease information using the EvoC ontology [23] for ESTs/cDNAs or direct parsing of CAGE/SAGE databases as explained in Materials and Methods. A gene was considered in the entropy calculation only if it had at least two alternative isoforms supported by at least 10 different transcripts from three separate libraries, thus a total of at least 20 transcripts mapped to each gene considered. In order to measure isoform entropy changes in a disease/normal tissue pair, we required that at least 50 genes and 100 isoforms were found expressed in both the normal and disease tissues. By considering only isoforms that were observed in both states, we excluded from our analysis spurious isoforms that are prevalent in many cancer EST libraries [24].

**Figure 2:**
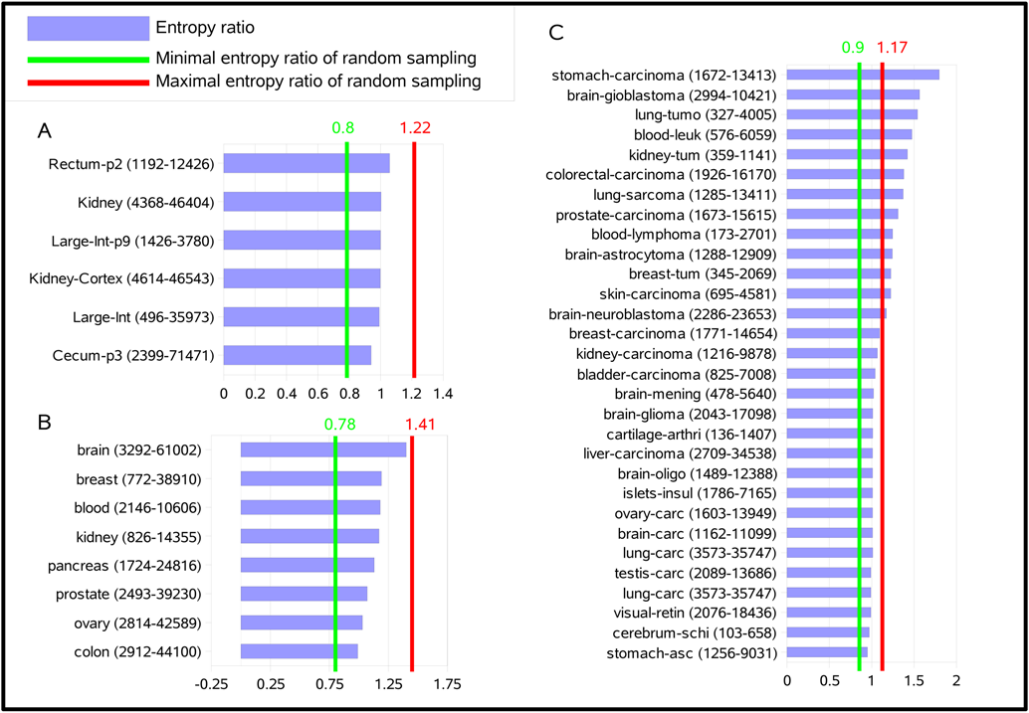
Ratio of average isoform entropy in cancer versus normal tissues. A value of 1 indicates that average entropy per gene in cancer tissue  =  average entropy per gene in normal tissue. The first number in parentheses corresponds to the number of genes that were used to calculate entropy gains, and the second corresponds the total coverage in ESTs/cDNAs/SAGEs for the diseased and normal tissue types. Only tissue types for which at least 50 genes and 100 isoforms were available to measure the entropy ratio are shown. (A) alternative initiation. (B) Alternative polyadenylation. (C) Alternative splicing.

We define the entropy ratio of a gene as the ratio of the entropy of this gene in the disease to the entropy of the same gene in the normal tissue. The entropy ratio of a disease/normal tissue pair is the average of the entropy ratios of all genes available in this tissue pair. Figure 2 presents entropy ratios for different diseases with respect to alternative initiation (A), polyadenylation (B) and splicing (C). An entropy ratio of one means that isoform entropy does not vary between disease and normal tissue (thick line in Figure 2). To estimate significance boundaries, random assays were performed by dividing the average entropy of 1000 randomly picked genes from any disease/tissue state by that of another randomly picked set of 1000 genes from any other disease/tissue state and repeating this process 10,000 times. This process was performed independently on the three isoform datasets. Values for the highest and lowest percentile are represented by red and green vertical lines, respectively.

Entropy ratios for alternative initiation and polyadenylation did not ever exceed the significance boundaries (Figure 2A and 2B) in the 6+8 cancer/normal tissue pair studied. This suggests that expression of alternative polyadenylation and initiation isoforms does not present large scale alterations in cancer. Alternative splicing however was quite different with 24 of the 27 cancer tissues studied showing a higher level of entropy than their normal counterpart (Figure 2C and Table S1). This entropy gain was highly significant in 13 cases, suggesting that the expression of splicing isoforms is strongly disrupted in certain cancers. In none of the 27 cases studied did the normal tissues show significantly higher entropy than disease tissues, and none of the three non-cancer diseases (arthritis, ascites and schizophrenia) presented a significant entropy change between normal and disease tissues.

The observed entropy bias is not imputable to sampling differences in normal and cancer libraries. The number of ESTs/cDNAs used to calculate entropy did not differ significantly between normal or disease tissues (Table S1), mainly due to the fact that we considered only isoforms that are expressed both in disease and normal tissues. Furthermore, Pearson's correlation tests (Table S1) showed no relationship between the entropy ratio and differences in the numbers of ESTs/cDNAs between normal and disease tissues (P = 0.28) or between the entropy ratio and the total size of libraries (P = 0.12). The observed gain in entropy can therefore not be attributed to a size effect of cancer EST libraries.

### Splice Factors Are Over-Represented Among Splice-Impaired Genes

In the ten most disrupted cancer tissues, splicing entropy gains were caused by 16 to 258 significantly disrupted genes, or 30%–68% of the gene set available for entropy calculation in these tissues. This suggests that splicing perturbation is caused by factors that regulate multiple genes at the same time. Sets of splice-disrupted genes from different tissues show little overlap therefore we cannot isolate a list of genes displaying a generally higher rate of splicing disruption. However, a clear functional trend appears when high entropy gain tissues are pooled together. In the ten cancer tissues that displayed the highest gain in splicing entropy (from stomach/carcinoma to brain/astrocytoma, Figure 2), we analyzed all genes showing a splicing entropy gain (414 genes) for functional enrichment. Interestingly, the most over-represented terms among splice-disrupted genes either contain “RNA splicing” or are higher level terms that incorporate RNA splicing (Table 1). The “RNA splicing” class mostly comprises splice factors. This suggests that splicing alterations in a few key splice factors could be involved in the more extensive splicing disruption observed in the high entropy-gain tissues. This enrichment is observable only after cancer tissues are pooled, which means the number of disrupted splice factors in a single disease is low. A total of 13 splice factors show a significant increase in splicing entropy in the cancer tissues studied (Table S2). Most are constitutive splice factors, only three (TRA2B, U2AF1, SF3A2) being involved in alternative splicing regulation.

**Table 1:** Gene Ontology term biases for genes with entropy gain in high-entropy cancer tissues, as measured using the Gene Ontology Toolbox [38].

Enriched GO Term	P-value
Cellular physiological process	1.55E-10
RNA metabolism	2.87E-10
RNA processing	3.48E-08
mRNA metabolism	4.74E-08
RNA splicing, via transesterification reactions	8.89E-08
RNA splicing with bulged adenosine as nucleophile	8.89E-08
Nuclear mRNA splicing, via spliceosome	8.89E-08
Primary metabolism	1.35E-07
RNA splicing	1.69E-07

Enrichment is measured relatively to all genes in the genome.

Splice factors are subject to alternative splicing at higher rates than average genes: 72% of the 58 annotated splice factors in Gene Ontology [22] have at least one alternative splice form in the ATD database [25], with an average of 5.4 isoform per gene, compared to 62% alternative splicing and 3.4 isoform per gene in the total ATD gene set. To test whether this bias could explain the over-representation of splice factors among disrupted genes in the high entropy gain cancers, we performed the same GO-term analysis among splice-disrupted genes in the ten disease categories displaying the lowest entropy gain. We could not observe any functional bias in this gene set (not shown). Therefore, splicing deregulation of splice factors is a hallmark of tissues where overall splicing is deregulated. This again designates misplicing of splice factors as a possible cause of wider splicing disruption in these tissues.

### Splicing Entropy Gain Is Correlated to Proliferation Signature

**Figure 3:**
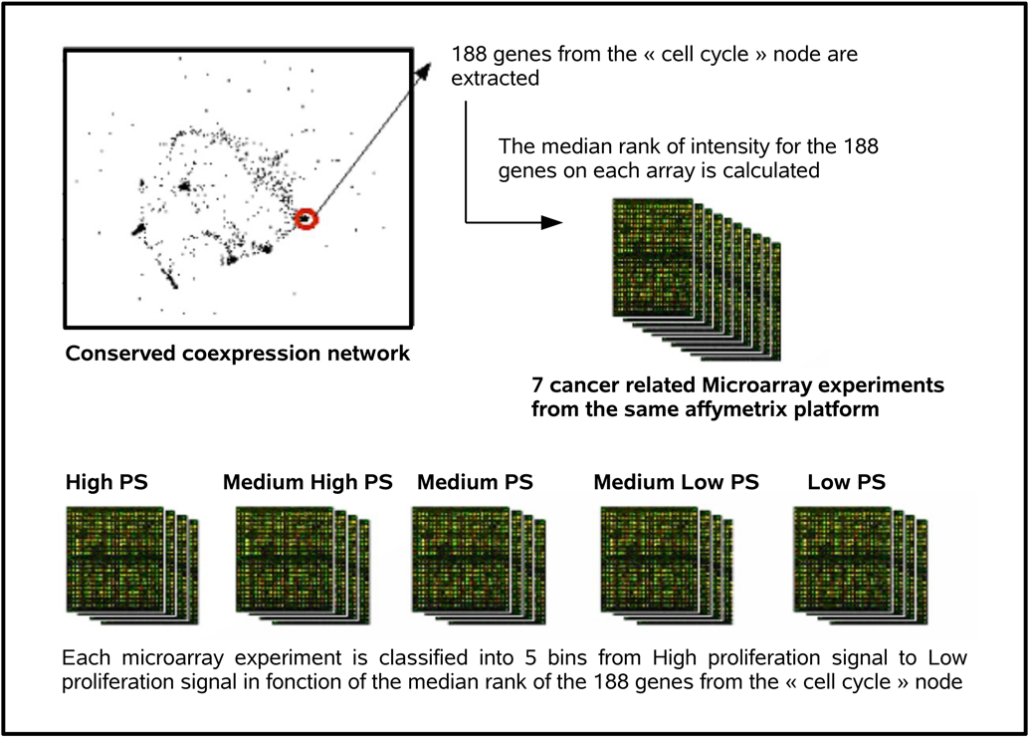
Meta-analysis method to obtain proliferative indices of cancer samples in microarray experiments. The 188 genes of the “cell cycle” cluster in the conserved coexpression network identified by Stuart et al. [26] were extracted. Each of the 3787 cancer-related samples was classified in one of 5 separate bins of same size in function of the average expression level of these 188 genes. The high proliferation signature bin (High PS) corresponds to the 20% of samples that have the highest mean expression level of the 188 genes; the lowest proliferation signature bin (Low PS) corresponds to the 20% of samples that have the lowest mean expression level of the 188 genes.

**Figure 4:**
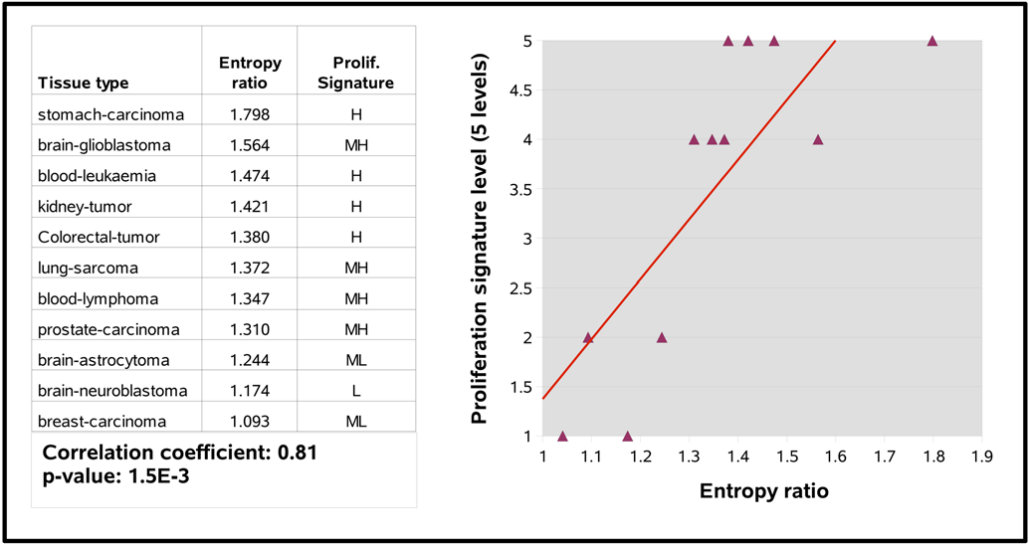
Correlation between the proliferation signature of different cancers and their splicing entropy ratio.

Although tumors are diverse and heterogeneous, they all share the key ability to proliferate at a higher level than normal tissue and this despite the very tight control that the organism usually exerts on cell proliferation. To test potential links between disordered isoform expression and higher levels of proliferation, we classified the cancer types that deregulate the splicing mechanism (Figure 2C) in function of their proliferative potential. To evaluate proliferation, we extracted the 188 genes from the “cell cycle” module of Stuart et al. [26], a cluster of coexpressed genes shown to be enriched in elements that are overexpressed in highly proliferative cells and whose high expression is a marker of entry into the cell cycle [27]. We manually verified each of these 188 genes (Table S3) and confirmed that 92 were shown to be specifically over-expressed during one of the replicative phases of the cell cycle and another 17 bore significant proof of being over-expressed in proliferating cells. We thus used a high expression of these markers as a surrogate for a high level of proliferation. In order to obtain a “proliferation index” of cancer samples, we computed the median expression level of the 188 markers in each of 3787 published Affymetrix microarray experiments performed on cancer samples [28]. Samples were then binned into five categories from low to high proliferation, as shown in Figure 3. To relate proliferation levels to splicing entropy results, we considered only microarray samples that contained the exact same keywords as disease tissues in Figure 2C. Results are shown in Figure 4. Cell proliferation, as measured from the expression of cell cycle genes, is significantly correlated to splicing entropy gains.

**Figure 5:**
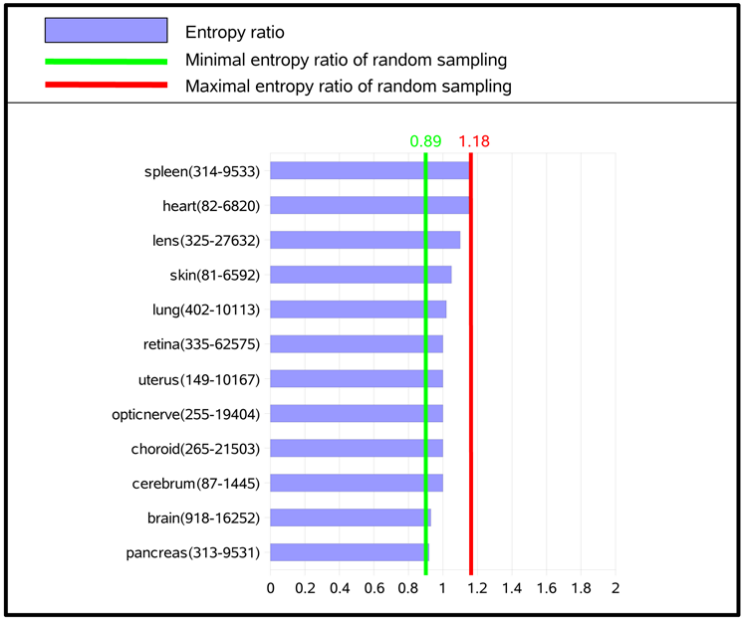
Ratio of average isoform entropy in fetus versus adult tissues for alternative splicing. The first number in parentheses corresponds to the number of genes that were used to calculate entropy gains, and the second corresponds to the total coverage in ESTs/cDNAs for the fetal and adult tissue types.

This observation led us to question the possible correlation between splicing entropy and cellular proliferation in a non-pathological context. We compared the splice isoform entropy of foetal and adult tissues in the same manner we compared disease and normal tissues (Figure 5). While foetal tissues are expected to present higher levels of proliferation than their adult counterparts, we could not observe any significant entropy gain in foetal tissues. This suggests the higher isoform entropy observed in highly proliferating cancers is only indirectly related to proliferation (proliferation indices of foetal tissues could not be obtained due to insufficient foetal microarray data).

## Discussion

While previous studies of cancer-related splicing alterations have focused mainly on the discovery of “aberrant” splice variants, we looked instead at changes in the balance of variants expressed in both healthy and cancer tissues. This new perspective enabled us to characterize another kind of splicing disorder in which splice variant expression profiles are significantly flattened in tumors. While isoforms from the same gene are usually differentially expressed in a given tissue, with clear minor and major forms, these expression differences are reduced in cancer and this leads to a raise of isoform entropy. Although controlled over/under-expression events may in principle produce a flattened profile, we find unlikely that the generalized entropy gain observed in cancer could result from a combination of multiple controlled changes in isoform expression. The entropy gain is more likely a sign of a general loss of regulation involving widespread, non-specific perturbations of alternative splicing. We did not observe such cancer-related disorders in alternative transcription initiation and alternative polyadenylation, the two other processes associated with expression of disease-specific isoforms.

**Figure 6:**
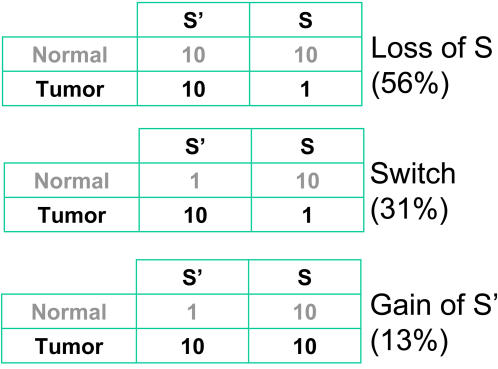
Classification of cancer-specific splice events as proposed by Xu and Lee [29]. Three typical cases of cancer-specific events are shown. Numbers are EST counts supporting each splice form. S: putative normal splice form; S': putative cancer-specific splice form. Percentages in parenthesis indicate the proportion of overall cancer-specific events that belong to each category according to [29].

Previous efforts to identify cancer-specific splice forms, either through EST analysis or experimental means, have mostly ignored non-specific, large-scale disruptions. An exception is the study by Xu and Lee [29] which sought splice forms with statistically significant expression changes between normal and tumor EST libraries. In that sense, these authors were looking for events that would cause an entropy reduction, not an entropy gain. However, they also discussed the impact of unspecific disruptions and analyzed expression patterns that may lead to cancer-specific isoforms (Figure 6). The most frequent patterns leading to cancer-specific events were the loss of a normal isoform S, and the switch in expression between normal (S) and cancer-specific (S') isoforms. A general entropy gain would go against the occurrence of such events, which makes these patterns even more interesting on a background of entropy gain. Contrarily, the “gain of S'” category is directly correlated to a rise of entropy (*i.e.* the “tumor” situation has higher entropy). Therefore, in a context of general entropy gain, events of the “gain of S'” category, even when statistically significant, could merely reflect the wider splicing disruption and should be considered with caution. Xu and Lee rightly noted that this category, which produces only a small fraction of cancer-specific splice forms, may be related to a loss of splicing specificity in tumors.

**Figure 7:**
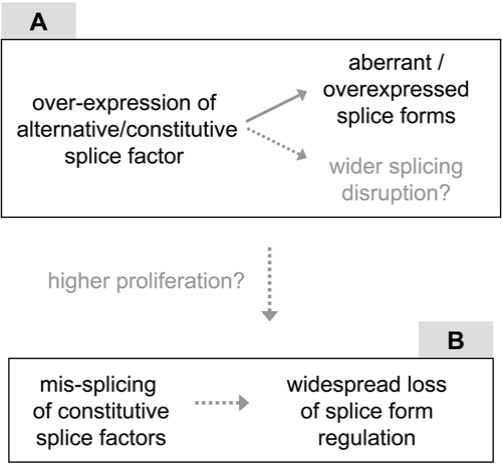
Models for mechanisms leading to specific or non-specific expression of splice isoforms in cancer tissues. Dotted arrows: hypothetical links. Box A: Known *trans* effect in which change in splice factor activation results in specific changes in the expression levels of several splice variants. Box B: Possible alternative mechanism in which disruption of SR protein splicing induces a wider deregulation of splice isoform expression. The dotted arrow between boxes indicates a possible link between specific and non-specific splicing disruption that may occur preferentially in proliferating tumors.

There is now ample evidence that changes in splice factor expression, due for instance to kinase activation [14], disrupt splicing patterns in tumors [16], [18]–[20],[30],[31]. Figure 7, box A presents the most common of these effects, where an up-regulated splice factor causes expression of a rare or aberrant splice form. Splice factors previously analyzed for such dysfunctions include SF2/ASF, U2AF-65, SFRS2, SFRS3, SRm160, hnRNP A1/A2, and TRA2-β, all acting both in alternative and constitutive splicing. Although these factors may potentially target many genes, studies have focused on specific targets such as CD44 and have not examined more widespread splice defects. The splicing disruptions that we observed apparently affect a larger number of transcripts and are characterized by a loss of splice form regulation. Although this phenomenon might occur as a byproduct of the above mechanism, its association with the mis-splicing of splice factors, prevalently of the constitutive type, leads us to postulate a second process (Figure 7, box B) in which mis-splicing of general splice factors would cascade into a wider splicing disruption and entropy gains. Among the 13 splice factors that displayed splicing disruptions in our study, two were already known to regulate their own splicing: SFRS3 and TRA2-β [15],[28]. In each case, overexpression of the splice factor activated the inclusion of stop codon-containing exons [15],[28] producing transcripts subject to nonsense-mediated decay [32],[33]. Both genes have additional isoforms that are not NMD-prone (Figure S1) and may contribute to the mis-splicing of other genes.

A possible link between the two pathways in Figure 7 naturally comes to mind when considering that a change in splice factor expression in pathway “A” could alter the splice variant balance of other splice factors in pathway “B”. This transition may occur preferentially in highly proliferating tumors, where we observed the strongest splicing disruption. Splicing perturbation is knowingly correlated to proliferation [31] however no causal relationship between these events has been identified yet. Perhaps the splicing mechanism has trouble in trying to keep up with the accelerated pace of cell proliferation or a general disorder in splicing is causing failure in the regulation of cell cycle. Independently of any mechanistic hypothesis, splicing entropy measures show that widespread splicing disruption may be prevalent in most cancer tissues. In such a context of high splicing entropy, therapeutic avenues involving the reprogrammation of mis-spliced isoforms [34] would have a limited interest. As already recognized in different studies [35],[36] splice factors or their regulatory machinery may turn out as better therapeutic targets.

## Materials and Methods

### Alternative Transcript and Expression Data

Transcripts and expression data for each type of transcriptional variation (initiation, splicing, polyadenylation) were obtained from the following sources.

Alternative initiation isoforms were obtained from the CAGE Basic/Analysis databases at http://fantom31p.gsc.riken.jp/cage_analysis/hg17/. This database classifies 3,106,472 CAGE tags into 450,228 transcription clusters (TC) further grouped into 32,351 transcription units (TU). TCs and TUs are two operationally defined units proposed in FANTOM3 [1] used to characterize promoters and genes respectively. We considered only those TCs that bore proof from at least 3 different CAGE libraries and 10 transcripts. These TCs were downloaded from the RIKEN website as well as the mappings of CAGE transcripts to these TCs in a given tissue type. This allowed us to create a relational database in which each TC could be queried to display its mapped CAGEs in each tissue type and the TU to which it belongs. For each normal/disease tissue pair we could therefore query a list of TCs common to both tissue types, link these TCs to their specific TUs and obtain the number of CAGEs mapped to a each of these TCs from the normal tissue library and from the disease tissue library.

Alternative polyadenylation isoforms were downloaded from the EBI ATD database, Human Release 1 (31 May 2005) [25] at http://www.ebi.ac.uk/atd/humrel1.html. Here, we only considered poly(A) sites located in the 3′-most exon of the gene because poly(A) sites located in upstream exons can belong to different splice forms. Since alternative splicing and polyadenylation can interfere [37], such events cannot be safely attributed to either phenomena. Again, each alternative polyadenylation event had to be supported by three different cDNA libraries and 10 transcripts, giving a total of 206,138 transcripts mapped to 13,367 poly(A) sites for 4400 genes. These 13,367 poly(A) sites were downloaded from the ATD website as well as the mapping of ESTs, cDNAs and SAGES to these isoforms. cDNA and EST transcripts were then linked to the eVOC 2.6 ontology through their Genbank accession identifiers and SAGE transcripts were manually parsed for simple tissue descriptors that were identical to eVOC 2.6 ontology terms (39 descriptors from the Gene Expression Omnibus [27]). This allowed us to create a relational database in which each poly(A) isoform could be queried to display its mapped transcripts in each tissue type and the Ensembl gene ID to which it belonged. For each normal/disease tissue pair we could therefore query a list of poly(A) isoforms common to both tissue types, link these isoforms to their specific Ensembl gene identifier and obtain the number of transcripts mapped to a each of these isoforms from the normal tissue library and from the disease tissue library.

Alternate splice isoforms were also downloaded from the EBI ATD database, Human Release 1. Again, 3 separate libraries and 10 transcripts were required to establish a splice form. Transcripts that mapped to multiple isoforms were excluded from the study bringing the total number of transcripts/isoforms/genes in the database from 808845 / 52742 / 14791 to 444799 / 47308 / 12281. These 47,308 alternative splice sites were downloaded from the ATD website as well as the mapping of ESTs and cDNAs to these isoforms. cDNA and EST transcripts were then linked to the eVOC 2.6 ontology through their Genbank accession identifiers. This allowed us to create a relational database in which each alternative splicing isoform could be queried to display its mapped transcripts in each tissue type and the Ensembl gene ID to which it belonged. For each normal/disease tissue pair we could therefore query a list of splicing isoforms common to both tissue types, link these isoforms to their specific Ensembl gene identifier and obtain the number of transcripts mapped to a each of these isoforms from the normal tissue library and from the disease tissue library.

### Expression of “Cell Cycle” Genes and Proliferation Categories

Cell-cycle specific genes were extracted from the conserved co-expression network defined by Stuart et al. [26] and available for download at http://cmgm.stanford.edu/kimlab/multispecies. A matrix of gene-gene Euclidean distances was computed and used for hierarchical clustering using R software. The tree obtained was then split into several groups by specifying a cutoff height of 10. All genes in the “cell cycle” cluster were extracted and their respective Locuslink ID used for annotation.

Microarray expression data was obtained from the Gene Expression Omnibus [28] selecting Affymetrix GPL96 platform (8340 different samples). We parsed microarray sample descriptions for the presence of any EvoC ontology keyword inherited from the top level term ≪neoplasia≫ and then manually checked to see if the description genuinely corresponded to a cancer-related experiment. From a set of 8340 microarray samples studied, 3787 samples corresponded to cancer-related microarray experiments. Proliferation categories were then attributed to each sample based on the median ranking (MR) of the expression level of the 188 genes from the cell cycle node, as follows: High proliferation : MR in the top 20% of the genes on array.; Medium-high proliferation : MR between top 20% and top 40% of genes on array; Medium proliferation : MR between the top 40% and top 60% of the genes on array; Medium-low proliferation: MR between bottom 20% and bottom 40% of genes on array; Low proliferation: MR in the bottom 20% of genes on array.

## Supporting Information

Figure S1 Alternative forms of splice factors TRA2B and SFRS3 in human, taken from the ASTD database, beta site (http://www.ebi.ac.uk/tc-test/astd/main.html). Major and NMD forms are indicated for each gene.
(1.57 MB TIF)

Table S1 Raw data from Figure 2 and correlation tests showing independance of entropy ratio to transcript coverage and number of genes tested.
(0.02 MB XLS)

Table S2 List of splice-disrupted splicing factors (high entropy gain in cancer).
(0.02 MB XLS)

Table S3 Detailed annotation of genes used to calculate proliferation level.
(0.14 MB XLS)
