# Supplementary figures and images for: Entropy Measures Quantify Global Splicing Disorders in Cancer
Source: PLoS Comput Biol. 2008 Mar 14;4(3):e1000011. doi: 10.1371/journal.pcbi.1000011 (PMC2268240; doi:10.1371/journal.pcbi.1000011)

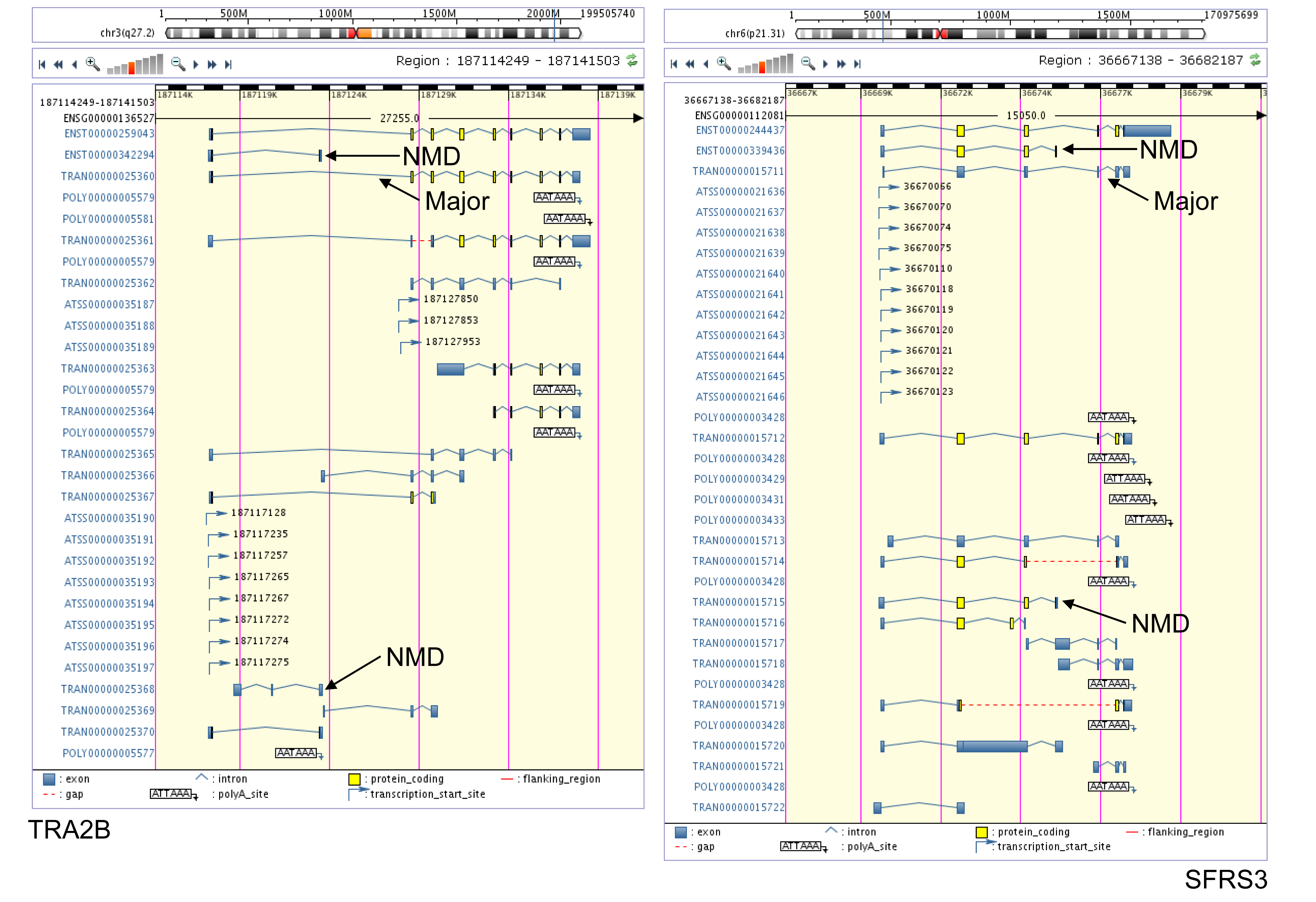

Supplement: Figure S1 — Alternative forms of splice factors TRA2B and SFRS3 in human, taken from the ASTD database, beta site (http://www.ebi.ac.uk/tc-test/astd/main.html). Major and NMD forms are indicated for each gene. (1.57 MB TIF) [file pcbi.1000011.s001.tif]
